# Mechanism of Action of Surface Immobilized Antimicrobial Peptides Against *Pseudomonas aeruginosa*

**DOI:** 10.3389/fmicb.2019.03053

**Published:** 2020-01-22

**Authors:** Muhammad Yasir, Debarun Dutta, Khondker R. Hossain, Renxun Chen, Kitty K. K. Ho, Rajesh Kuppusamy, Ronald J. Clarke, Naresh Kumar, Mark D. P. Willcox

**Affiliations:** ^1^School of Optometry and Vision Science, University of New South Wales, Sydney, NSW, Australia; ^2^Optometry and Vision Science, Optometry School, Aston University, Birmingham, United Kingdom; ^3^School of Chemistry, The University of Sydney Nano Institute, The University of Sydney, Sydney, NSW, Australia; ^4^School of Chemistry, University of New South Wales, Sydney, NSW, Australia

**Keywords:** antimicrobial peptides, surface immobilization, *Pseudomonas aeruginosa*, mode of action, membrane disruption

## Abstract

Bacterial colonization and biofilm development on medical devices can lead to infection. Antimicrobial peptide-coated surfaces may prevent such infections. Melimine and Mel4 are chimeric cationic peptides showing broad-spectrum antimicrobial activity once attached to biomaterials and are highly biocompatible in animal models and have been tested in Phase I and II/III human clinical trials. These peptides were covalently attached to glass using an azidobenzoic acid linker. Peptide attachment was confirmed using X-ray photoelectron spectroscopy and amino acid analysis. Mel4 when bound to glass was able to adopt a more ordered structure in the presence of bacterial membrane mimetic lipids. The ability of surface bound peptides to neutralize endotoxin was measured along with their interactions with the bacterial cytoplasmic membrane which were analyzed using DiSC(3)-5 and Sytox green, Syto-9, and PI dyes with fluorescence microscopy. Leakage of ATP and nucleic acids from cells were determined by analyzing the surrounding fluid. Attachment of the peptides resulted in increases in the percentage of nitrogen by 3.0% and 2.4%, and amino acid concentrations to 0.237 nmole and 0.298 nmole per coverslip on melimine and Mel4 coated surfaces, respectively. The immobilized peptides bound lipopolysaccharide and disrupted the cytoplasmic membrane potential of *Pseudomonas aeruginosa* within 15 min. Membrane depolarization was associated with a reduction in bacterial viability by 82% and 63% for coatings melimine and Mel4, respectively (*p* < 0.001). Disruption of membrane potential was followed by leakage of ATP from melimine (1.5 ± 0.4 nM) or Mel4 (1.3 ± 0.2 nM) coated surfaces compared to uncoated glass after 2 h (*p* < 0.001). Sytox green influx started after 3 h incubation with either peptide. Melimine coatings yielded 59% and Mel4 gave 36% PI stained cells after 4 h. Release of the larger molecules (DNA/RNA) commenced after 4 h for melimine (1.8 ± 0.9 times more than control; *p* = 0.008) and after 6 h with Mel4 (2.1 ± 0.2 times more than control; *p* < 0.001). The mechanism of action of surface bound melimine and Mel4 was similar to that of the peptides in solution, however, their immobilization resulted in much slower (approximately 30 times) kinetics.

## Introduction

Bacterial colonization and subsequent biofilm development on medical devices can lead to peri-implantitis and device failure (Papathanasiou et al., 2016). It is estimated that every person may use at least one implant in their lifetime (Gristina, 1987). The use of polymer-based implants is increasing and is contributing to the growth of the implant market that is expected to reach $33 billion by 2025 (Greenhalgh et al., 2019). Despite continuous improvements in device design, infections occur in 2 to 14% of implants (cardiovascular, orthopedic, neurosurgical, plastic surgical, contact lenses, and urinary catheters) (Costerton et al., 1999; Darouiche, 2001, 2004; Foxman and Brown, 2003; Zimmerli et al., 2004; Trampuz and Widmer, 2006; Kazemzadeh-Narbat et al., 2010). Implanted devices account for approximately 50% of all hospital infections (Darouiche, 2004; Bryers, 2008). Importantly, 80% of such infections are associated with bacterial biofilms, which impair the efficacy of antibiotic treatment by increasing antibiotic resistance of cells in the biofilms by 10–1000 times (Lewis, 2001; de Carvalho, 2007; Monteiro et al., 2009).

*Pseudomonas aeruginosa* frequently colonizes catheters and other medical devices (Brouqui et al., 1995; Oliver et al., 2000; Laverty et al., 2014; Rasamiravaka et al., 2015) and accounts for 10–20% of all hospital-acquired device and non-device related infections (Ramos et al., 2013). Approximately 2 million patients are infected annually by *P. aeruginosa* and 90,000 of them die from these infections (Cross et al., 1983). Many of these infections are associated with catheterization and intubation (Cross et al., 1983). The ability of *P. aeruginosa* to thrive in different ecological niches and the emergence of antimicrobial resistance can lead to chronic infections (Morrison and Wenzel, 1984; Laverty et al., 2014). Thus, there is a need for the development of antimicrobial biomedical devices which can resist *Pseudomonas* attachment and ultimately reduce infections (Hilpert et al., 2009; McCloskey et al., 2014).

Antimicrobial surface coatings have emerged as promising approaches to control medical device-mediated infections (Matl et al., 2008). Antibiotics such as cefazolin, rifampin, vancomycin, and polymyxin B retain their antimicrobial activity upon surface immobilization (Campoccia et al., 2010; Mohorcic et al., 2010; Palchesko et al., 2011), but the use of these antibiotics may result in the development of antibiotic resistance (Landman et al., 2008). Silver (Jones et al., 2006) quaternary ammonium compounds (Ravikumar et al., 2006), salicylic acid (Bryers et al., 2006) or polymeric substances (Siedenbiedel and Tiller, 2012) are highly effective *in vitro*, but use of these chemicals can induce toxicity *in vivo* (Ramstedt et al., 2009). Loss of activity and the inability to sterilize surfaces once antimicrobials are covalently bound to devices are also problems associated with antimicrobial coatings (Vasilev et al., 2009; Townsend et al., 2017).

Antimicrobial peptides (AMPs) are promising bioactive molecules (Brogden and Brogden, 2011) which are highly biocompatible and relatively resistant to the development of bacterial resistance (Gordon et al., 2005; Willcox et al., 2008; Costa et al., 2011). Several AMPs have been successfully covalently immobilized on a variety of materials such as contact lenses, glass, titanium oxide, resin beads, silicone surfaces (Bagheri et al., 2012; Shalev et al., 2012; Li et al., 2014; Chen et al., 2016; Nie et al., 2017; Dutta et al., 2018a). However, the bactericidal mechanism of these immobilized AMPs is yet to be fully elucidated, and it seems unlikely that they could act intracellularly as some free AMPs have been observed to act. (Le et al., 2017).

Melimine (TLISWIKNKRKQRPRVSRRRRRRGGRRRR) is a cationic chimeric peptide of two naturally occurring peptides melittin and protamine (Willcox et al., 2008). Melimine has a wide spectrum of activity targeting Gram-negative and Gram-positive bacteria (including methicillin resistant *S. aureus* MRSA, and multi-drug resistant *P. aeruginosa*), fungi and protozoa such as *Acanthamoeba* (Willcox et al., 2008; Dutta et al., 2013). Bacteria do not develop resistance against melimine when exposed at sub-MIC for 30 consecutive days (Willcox et al., 2008). Moreover, it is not cytotoxic at well above active concentrations (Willcox et al., 2008; Dutta et al., 2013). Melimine retains its antimicrobial activity when bound to polymers and titanium (Willcox et al., 2008; Dutta et al., 2013; Chen et al., 2016, 2017).

Phase I and Phase II clinical trials registered with the Australian New Zealand Clinical Trial Registry (trial ID ACTRN 12613000369729) revealed that melimine-coated lenses did not induce conjunctival redness and or fluorescein staining of the human cornea during wear (Dutta et al., 2014). However, melimine-coated lenses produced corneal staining in some subjects (Dutta et al., 2014).

A derivative of melimine called Mel4 (KNKRKRRRRRRGGRRRR) is active against *P. aeruginosa* when it is immobilized on surfaces (Chen et al., 2017). It is non-cytotoxic to mammalian cells *in vitro* (Dutta et al., 2013), in animal model studies and in human clinical trials (Chen et al., 2017; Dutta et al., 2018b). Mel4-coated contact lens did not produce any signs of ocular irritation, redness or corneal staining during a Phase 1 trial (Dutta et al., 2017). Phase II/III clinical trials, registered with the Australia and New Zealand Clinical Trial Registry (ACTRN1261500072556), showed that Mel4-coated lenses did not elicit ocular discomfort or change the ocular surface physiology (including corneal staining) during extended contact lens wear for 14 days. Furthermore, these Mel4-coated contact lenses reduced the incidence of corneal infiltrative events by 50% (Dutta et al., 2018a; Kalaiselvan et al., 2018).

The mechanism of action of both AMPs in solution involves interaction with lipopolysaccharide, permeabilization of inner membrane and release of cellular contents such as ATP and nucleic acid leading to lysis of pseudomonal cells (Rasul et al., 2010; Yasir et al., 2019). How surface bound melimine and Mel4 interacts and kills bacteria and whether their antimicrobial activity after immobilization is similar to that when they are free in solution is unknown. Thus, the current study was designed to evaluate the mechanism of action of these surface-immobilized AMPs against *P. aeruginosa.*

## Materials and Methods

### Bacterial Strains and Growth Conditions

*Pseudomonas aeruginosa* strains 6294 and 6206 (isolated form microbial keratitis; 6294 an invasive strain containing the *exoS* gene and 6206 a cytotoxic strain containing the *exoU* gene) (Zhu et al., 2006), Paer1 (isolated from contact lens induced acute red eye, containing the *exoS* gene but not manifesting the associated invasive phenotype) (Zhu et al., 2006) and ATCC 19660 (human septicemia; a cytotoxic strain containing the *exoU* gene) (Pillar et al., 2000) were used in the current study.

Bacteria were grown overnight in Tryptic Soy Broth (TSB; Oxoid. Basingstoke, United Kingdom) to mid-log phase and cells were then washed with 5 mM HEPES (Sigma Aldrich, St. Louis, MO, United States) buffer (pH 7.2) containing 20 mM glucose. The OD_600__nm_ was adjusted to 0.05–0.06 (in HEPES buffer containing 1/1000 TSB to yield 1 × 10^7^ colony forming units (CFU/ml) upon retrospective plate counts on Tryptic Soy agar (TSA; Oxoid, Basingstoke, United Kingdom). All experiments were performed using HEPES buffer containing 1/1000 TSB except for membrane depolarization where the release of DiSC3-5 from cells was determined in HEPES buffer alone. All experiments were conducted three times in triplicate. Negative controls were bacterial cells incubated with process control and untreated uncoated surfaces.

### Covalent Attachment of Peptides

Melimine and Mel4 (≥90% purity) were synthesized by conventional solid-phase peptide protocols (Gongora-Benitez et al., 2013; Behrendt et al., 2016) and procured from Auspep Peptide Company (Tullamarine, VIC, Australia). Peptides were immobilized onto glass surfaces (including quartz glass slides for circular dichroism experiments) as described previously (Chen et al., 2009) using 4-azidobenzoic acid, UV irradiation and reaction with 1-[(3-dimethylamino)-propyl]-3-ethylcarbodiimide hydrochloride (EDC; Alfa Aesar, 65 mM; 5 mg/ml) in PBS for 1 h at room temperature. Finally, ABA–EDC functionalized surfaces were reacted with melimine or Mel4 (2 mg/ml in PBS) for 24 h at room temperature in a humidified chamber. Following incubation, peptide-coated coverslips were washed extensively in sterile PBS, and then resuspended in PBS and kept overnight with gentle shaking to remove any non-adhered peptide from the coverslips. Covalent attachment of peptides was confirmed following treatment with (2% v/v) sodium dodecyl sulfate (SDS) at 55°C for 1 h. After incubation in SDS samples were washed (five times) with milli Q water and subjected to X-ray photoelectron spectroscopy (see below). Hot SDS removes peptides which are loosely bound or attach via physical absorption to the surfaces (Bilek and McKenzie, 2010).

### Surface Characterization

The composition of peptide-coated glass surfaces was analyzed using a X-ray photoelectron spectrometer (ESCALAB220-iXL, VG Scientific, West Sussex, United Kingdom) equipped with monochromated Al Kα source (hv) (1,486.6 eV), source power (120 W). Vacuum pressure was set at ≤10–8 mbar. Three samples of each peptide-coated surfaces were analyzed and the mean value (±SD) was reported. The amount of AMP attached to glass coverslips was also quantified using amino acid analysis (Dutta et al., 2016a). The sum of all the amino acids derived from each glass cover slip was regarded as total amount of AMPs attached to the glass coverslips.

### Circular Dichroism (CD) Spectroscopy Analysis of Mel4

Circular dichroism spectroscopy analysis of surface-bound and free Mel4 in 10 mM Tris, pH 7.2 was performed using a JASCO – 1500 spectropolarimeter at 30°C using a Peltier thermostat controlled cell holder PTC-517. The spectra were obtained over a wavelength range of 190–250 nm, using a 1 cm path length quartz cell at continuous scanning mode with a response of 1 s with 0.2 nm steps, a bandwidth of 2 nm, and a scan speed of 100 nm/min. For surface-bound Mel4, data were acquired after inserting the peptide-conjugated quartz slide (38 mm × 9 mm × 1 mm; Pro Sci Tech, Thuringowa, Queensland, Australia) into the 1 cm quartz cell as previously described (Gao et al., 2012). An empty quartz slide of similar dimension was used as a control sample. For solution CD experiments, solutions were prepared at a constant peptide concentration of 2.12 nmole/ml. After CD data acquisition of Mel4 in solution or bound to quartz surface, 0.25 mM and 0.5 mM of either DMPC:DMPG (1:1 molar ratio) or DPPC lipid vesicles were pipetted into the quartz cell. To make the lipid vesicles, dimyristoylphosphatidylcholine (DMPC), dimyristoylphosphatidylglycerol (DMPG) or dipalmitoylphosphatidylcholine (DPPC; Avanti Polar Lipids, Alabama, United States) in chloroform were dried overnight using a stream of nitrogen gas to remove most of the chloroform, followed by the addition of 1 ml of 10 mM Tris, pH 7.2 buffer. DMPC and DMPG were mixed in a ratio on 1:1, and DPPC was used alone. The lipids were then sonicated in a water bath for a minimum of 30 min to ensure lipid vesicle formation followed by extrusion using a Mini-Extruder (Avanti Polar Lipids) in order to form unilammellar vesicles. For all samples, corresponding background samples without peptides were prepared for spectral subtraction. Four spectra for each condition were averaged to achieve an appropriate signal-to-noise ratio. Finally, each spectrum was corrected by subtracting the background from the sample spectrum. The web server CAPITO (CD Analysis and Plotting Tool^1^) (Wiedemann et al., 2013) was used to evaluate the CD spectra.

### Ability of the Antimicrobial Peptides-Coated Surfaces to Bind Lipopolysaccharides

A limulus amoebocyte lysate (LAL) assay was conducted to assess the interaction of surface bound AMPs with lipopolysaccharides (LPS) of *P. aeruginosa* using a chromogenic assay (Cape Cod, E. Flamouth, MA, United States) (Gustafsson et al., 2010). LPS (8 × 10^–4^ nmol/ml) from *P. aeruginosa* 10 (Sigma Aldrich, St. Louis, MO, United States) was dissolved in endotoxin free water (Sigma Aldrich) and incubated with surface bound AMPs at 37°C for 4 h. Following addition of LAL reagent, any decrease in OD_405__nm_ was measured and compared with control surface (without peptides), and results expressed as a percentage reduction compared to the control surface.

### Membrane Interactions

Cytoplasmic membrane depolarization caused by surface bound AMPs was determined (Hilpert et al., 2009; Yasir et al., 2019). Prior to exposure to surface bound AMPs, bacteria were labeled with 4 μM membrane potential sensitive dye DiSC3-5 (Sigma Aldrich) in the presence of 0.5 mM EDTA. Dye-labeled bacteria (200 μl) were incubated with peptide-coated and control surfaces. An increase in fluorescence due to release of DiSC3-5 from bacteria was recorded at an excitation wavelength of 622 nm and an emission wavelength of 670 nm. The effect of membrane depolarization on the viability of adhered cells was assessed by plate counts. Glass coverslips were washed with buffer, added to Dey-Engley neutralizing broth (D/E, Remel, United States), vortexed rapidly in the presence of a magnetic stirring bar to break up the glass and facilitate removal of bacterial cells, and the resulting suspension was plated on TSA containing 0.07% w/v phosphatidylcholine and 0.5% v/v Tween 80. The plates were incubated at 37°C for 16 h, and the number of live bacteria adhering were expressed as CFU/mm^2^.

Permeabilization of the cytoplasmic membrane by surface bound AMPs was evaluated (Rai et al., 2016). Bacteria (10^7^ CFU/ml) in the presence of Sytox green (Invitrogen, Eugene, OR, United States; 5 μM), were incubated with peptide-coated and uncoated surfaces. The surrounding buffer was drawn at regular intervals and fluorescence due to interaction of the Sytox green with DNA was determined spectrophotometrically at an excitation wavelength of 480 nm and emission wavelength of 523 nm. Cytoplasmic membrane damage was also assessed with the Live/Dead BacLight bacterial viability kit (Invitrogen, Eugene, OR, United States) (Chen et al., 2009).

### ATP and Nucleic Acid (DNA/RNA) Leakage

The effect of surface immobilized peptides on the leakage ATP was determined (Li et al., 2014). Aliquots (200 μl) of bacteria were incubated with peptide-coated and uncoated surfaces at 37°C for 4 h. Subsequently, supernatants were removed at 2 h intervals, centrifuged and analyzed for extracellular ATP using an ATP bioluminescence kit (Invitrogen, Eugene, OR, United States) according to manufacturer’s instructions. To determine whether release of ATP had any impact on bacterial viability, viable counts were performed as described above in the membrane depolarization assay.

The effect of surface bound peptides on the release of nucleic acid was also examined (Yasir et al., 2019). Bacteria suspended in HEPES were incubated with peptide-coated and uncoated surfaces at 37°C for 10 h. Aliquots (200 μl) were drawn at 2 h intervals, and filtered through 0.22 μm membranes (Merck, Tullagreen, Carrigtwohill, Ireland). The OD_260__nm_ of the filtrates was measured. After calculating the amount of each peptide bound to glass, the equivalent amount of free melimine (0.297 nmole/200 μl) and Mel4 (0.358 nmole/200 μl) were used to determine their effect on release of nucleic acid and compared with the amount released by surface immobilized peptides. The results were expressed relative to the initial OD_260__nm_.

### Statistical Analysis

Statistical analyses were performed using GraphPad Prism 7.02 software (GraphPad Software, La Jolla, CA, United States). Data from the three technical replicates obtained on each day were averaged, and then this data for the biological replicates obtained on each day (*n* = 3) was used for statistical analysis. The data for LPS interaction and confocal microscopy were analyzed for Gaussian distribution which showed these did not follow a Gaussian distribution and so this data were analyzed using non-parametric tests (Mann-Whitney *U*-test). Data obtained from LPS interactions were analyzed using one-way ANOVA with Bonferroni correction for multiple comparisons. Confocal microscopy data for the number of PI positive (red) cells were assessed with Kurskal-Wallis test and overall bacterial inhibition was analyzed using Dunnett’s test. The data (*n* = 3) obtained from time dependent interaction with the cell membrane (depolarization and permeabilization) and leakage of cellular contents (ATP and nucleic acid) were examined using two-way ANOVA with Tukey’s test for multiple comparisons. Correlations between membrane depolarization and bacterial death were examined using the Pearson correlation test. The average values of all the assays are reported. Statistical significance was set *p* < 0.05.

## Results

### Characterization of the Melimine and Mel4 Coated Surfaces

The percentage (%) composition of carbon (C), nitrogen (N), oxygen (O) and silicon (Si) concentrations of glass surfaces following attachment of melimine or Mel4 are given in Table 1. Change in the% composition of N and C are an indication of successful attachment of AMPs to the surface. There was an increase in % amide N on glass of 125% and 75% for melimine and Mel4, respectively, compared to the process control (Table 1). Similarly, % C increased by 106% for melimine and 59% for Mel4 surfaces compared to the process control. Amino acid analysis confirmed the attachment of melimine and Mel4 to coated glass surfaces (Table 1). SDS treatment removed only 11% of the nitrogen from melimine and 14% from Mel4 coated surfaces (Table 1) indicating that the vast majority of surface-associated peptides were covalently bound.

**TABLE 1:** Elemental composition and amount of peptide (amino acids) bound to glass surfaces.

Surfaces	% C	% *N* (% *N* after SDS wash)	% O	% Si	Amino acid (nmole)/glass coverslip
Melimine	21.8 ± 6.5	5.4 ± 1.8 (4.8 ± 0.7)	54.4 ± 5.4	18.4 ± 2.8	0.297 ± 0.010
Mel4	16.9 ± 2.1	4.8 ± 0.5 (4.1 ± 0.9)	55.2 ± 2.8	23.1 ± 1.1	0.358 ± 0.019
Process control control	10.6 ± 2.2	2.4 ± 1.2	62.0 ± 1.2	25.0 ± 2.1	0.060 ± 0.002
Blank	5.3 ± 1.2	1.0 ± 0.6	66.9 ± 0.8	26.7 ± 0.9	0.006 ± 0.006

### Circular Dichroism Spectroscopy Analysis of Mel4

**FIGURE 1:**
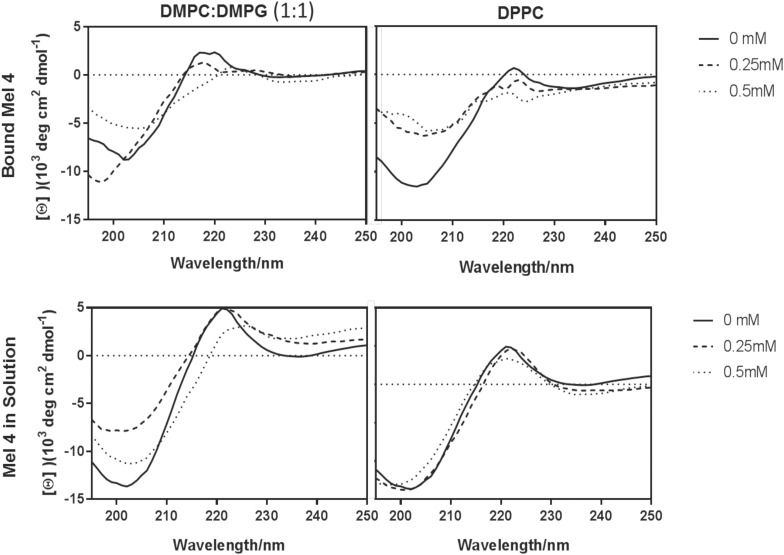
Lipid interactions of immobilized and free Mel4. Interaction with DMPC:DMPG (1:1) (left) and DPCC (right). The samples in DMPC:DMPG and DPPC were analyzed without lipids (solid), or in the presence of 0.25 mM (dashed), and 0.5 mM (dotted) lipids.

The minima in the spectra of free or bound Mel4 at ∼200 nm indicated that Mel4 mostly adopted a random coil. Mel4 did not interact with DPPC either when free in solution or when bound, as there was no change in the peak at 220 nm when exposed to increasing concentrations of these vesicle. On the other hand, the spectra for Mel4 interaction with 0.5 mM DMPC:DMPG in both the states showed a slight shift of the peak at 220 nm to the right, suggesting that the Mel4 peptide was becoming more ordered than that observed in the absence of this lipid mixture (Figure 1).

### Interaction With Lipopolysaccharides

**FIGURE 2:**
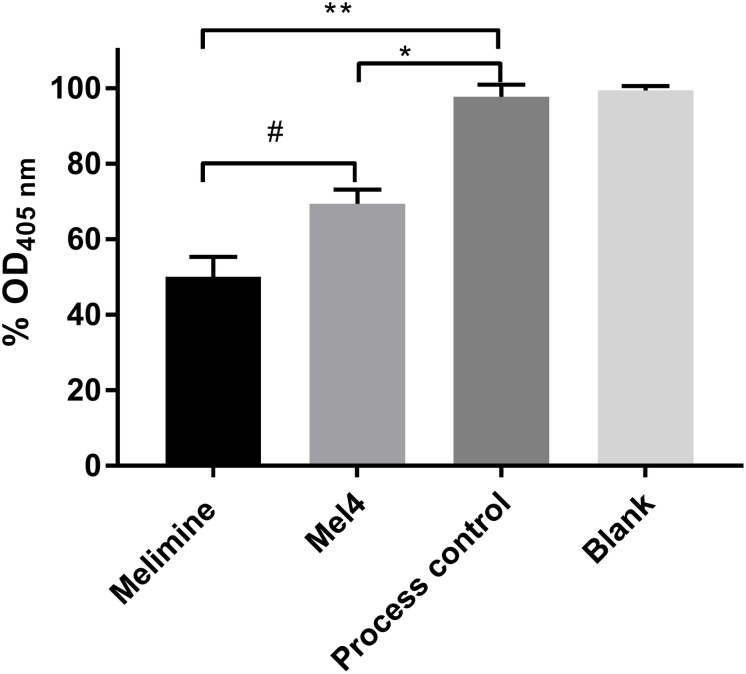
Interaction of surface immobilized peptides with lipopolysaccharides. An LAL assay was used to investigate the interaction of surface immobilized melimine or Mel4 with LPS of *Pseudomonas aeruginosa* 10. The OD_405__nm_ in (%) dropped significantly after incubation of LPS with peptides attached to glass. *p = 0.005 and **p < 0.001 compared to process control while ^#^*p* = 0.004 compared to Mel4.

Both surface immobilized AMPs bound LPS of *P. aeruginosa* 10. The melimine-coated surface lowered the OD_405__nm_ by 50 ± 5% (*p* < 0.001) while Mel4-coating reduced it by 31 ± 4% (*p* = 0.005) compared to control surfaces after 4 h of incubation (Figure 2). Melimine-coated surfaces bound significantly more LPS than Mel4-coated surfaces (*p* = 0.004).

### Membrane Interactions

All the subsequent results show data for *P. aeruginosa* 6294 only. Data for all other strains are presented in Supplementary Tables S1–S7. Both the surface immobilized peptides had identical modes of action against all four strains of *P. aeruginosa.*

**FIGURE 3:**
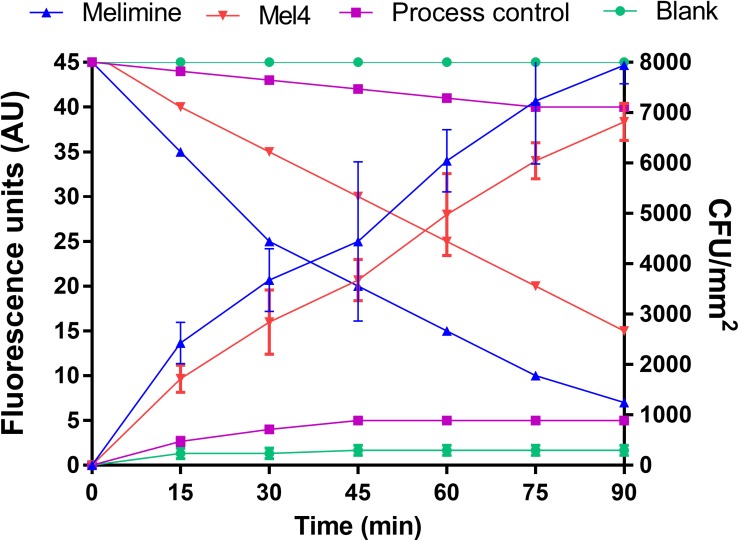
Cell membrane depolarization. Cell membrane depolarization of *P. aeruginosa* 6294 as assessed by the release of the membrane potential sensitive dye DiSC3-5, measured spectroscopically at 622_nm_ excitation and 670_nm_ emission wavelengths, and corresponding bacterial cell death as determined by plate counts. Each symbol represents data curve for increase in fluorescence (on the left *y* axis) and for reduction in bacterial count CFU/mm^2^ (on the right *y* axis). Data are presented as means (±SD) of three independent repeats performed in triplicate.

Both the surface bound AMPs led to measurable membrane depolarization in a time dependent manner as assessed with the release of DiSC3-5. The increase in fluorescence started after 15 min incubation and continuously increased until 90 min for both melimine and Mel4 coated surfaces (Figure 3). This was highly correlated with rapid decrease in the viability of surface attached bacteria (*R*^2^ ≥ 0.975). Following 90 min incubation, melimine and Mel4-coated surfaces killed 82% and 63% surface attached bacteria, respectively (*p* < 0.001, Figure 3).

**FIGURE 4:**
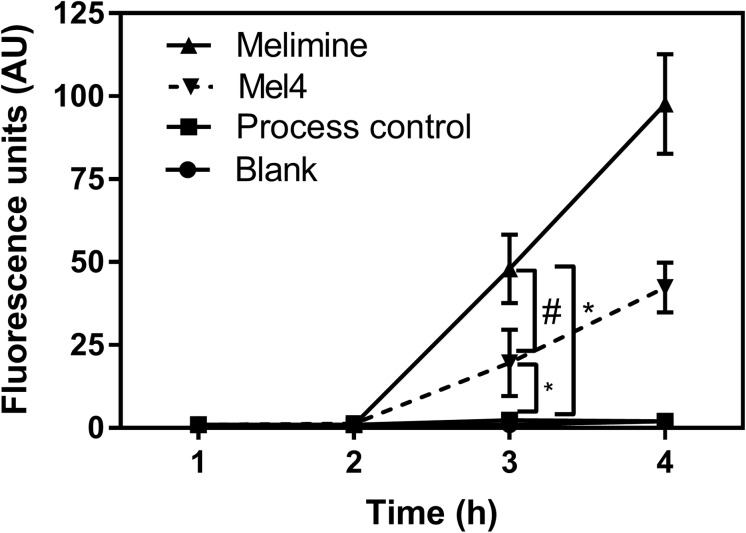
Inner membrane permeabilization. Inner membrane permeabilization of *P. aeruginosa* 6294 by surface attached melimine and Mel4 was determined using the membrane impermeable dye Sytox green. Fluorescence intensity due to interaction of Sytox green with DNA following incubation with peptides attached to surfaces was measured spectroscopically at 480_nm_ excitation and 522_nm_ emission wavelengths. *p < 0.001 compared to process control while ^#^*p* = 0.007 compared to Mel4. Error bars represent means (±SD) of three independent repeats in triplicate.

**FIGURE 5:**
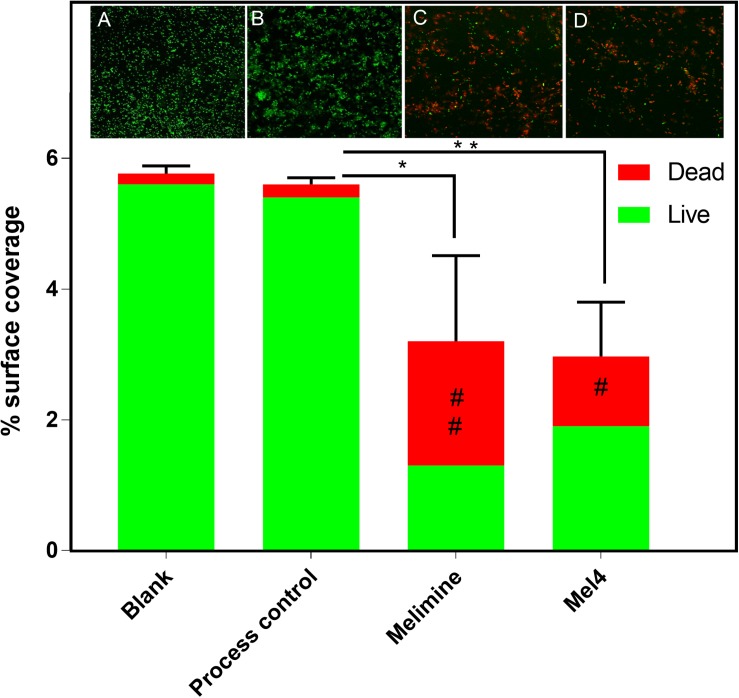
Fluorescence microscopy of bacteria adhered to glass surfaces. Bacterial cells with intact membranes stained green with Syto-9 while bacteria with permeabilized membrane stained red with propidium iodide. Representative images of *P. aeruginosa* 6294 captured with 40× objective following incubation for 4 h with **(A)** blank glass, **(B)** process control, **(C)** melimine coated, and **(D)** Mel4 coated surfaces. Image analysis of the percentage surface area covered by stained bacteria was carried out with ImageJ software. Percentage area covered by live bacteria is represented as green bars and the area covered by dead bacteria represented as red bars. *p = 0.047, **p = 0.032, ^#^*p* = 0.073, and ^##^*p* = 0.025 compared to process control. Error bars represent means of (±SD) of three independent repeats in triplicate.

Fluorescence intensity of Sytox green increased significantly after 3 h of incubation with both peptide-coated surfaces (*p* < 0.001; Figure 4). Melimine produced significantly higher fluorescence than Mel4 at 3 h (*p* = 0.007) and 4 h (*p* < 0.001). There was an increase in numbers of bacteria with permeabilized membranes (PI stained) on melimine and Mel4 coated surfaces compared to the controls after 4 h analyzed by LIVE/DEAD stains (Figure 5). Melimine-coated surfaces resulted in 59 ± 41% (*p* = 0.025) and Mel4-coated surfaces resulted in 36 ± 28% (*p* = 0.0736) of cells staining with PI after 4 h incubation (Figures 5C,D). Control surfaces resulted in less than 4% PI stained cells (Figures 5A,B). Moreover, melimine and Mel4 coated surfaces inhibited bacterial attachment by 43 ± 27% (*p* = 0.0475) and 47 ± 16% (*p* = 0.0328) compared with the process control (Figure 5). There was no significant difference in inhibition of bacterial adhesion between the melimine and Mel4 coated surfaces (*p* = 0.939).

### Leakage of ATP and Nucleic Acids (DNA/RNA)

**FIGURE 6:**
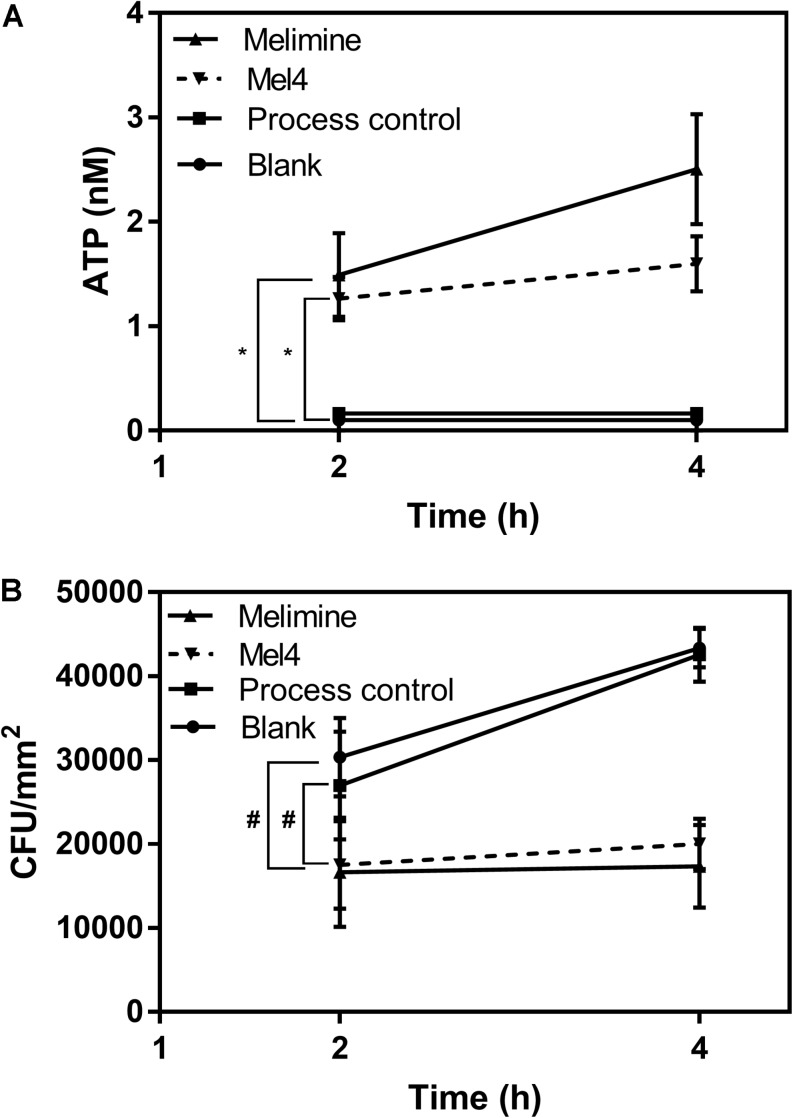
Leakage of ATP. **(A)** The leakage of ATP from *P. aeruginosa* 6294 following incubation with surface attached melimine, Mel4, process control or blank glass. *p ≤ 0.033 compared to process control. **(B)** Corresponding number of viable *P. aeruginosa* 6294 reduced by peptides attached surfaces. ^#^*p* ≤ 0.033 compared to process control. Error bars represent means (±SD) of three independent repeats in triplicate.

Release of ATP started after 2 h incubation with both peptide-coated surfaces (Figure 6A). Melimine (1.5 ± 0.4 nM) and Mel4 (1.3 ± 0.2 nM) coated surfaces resulted in the release of significantly more ATP than process control or blank surfaces (*p* < 0.001). The concentration of extracellular ATP increased to 2.5 ± 0.5 nM and 1.6 ± 0.3 nM for melimine and Mel4-coated surfaces, respectively, after 4 h of incubation (*p* < 0.001). The ATP concentration in the surrounding fluid of control surfaces did not change during the entire length of the experiment (Figure 6). Release of ATP was associated with a reduction in viability of surface attached bacteria. At 2 h, melimine and Mel4-coated surfaces killed 38 ± 24% and 35 ± 19% bacteria, respectively, compare to process control surfaces (*p* ≤ 0.033, Figure 6B). There was a higher reduction of 63 ± 16% for melimine and 53 ± 7% for Mel4 compared to process control surface after 4 h (*p* ≤ 0.006; Figure 6B). The bactericidal effect of both AMP-coated surfaces was similar and there was no significant difference between the two AMP-coated surfaces after 4 h incubation (*p* ≥ 0.697).

**FIGURE 7:**
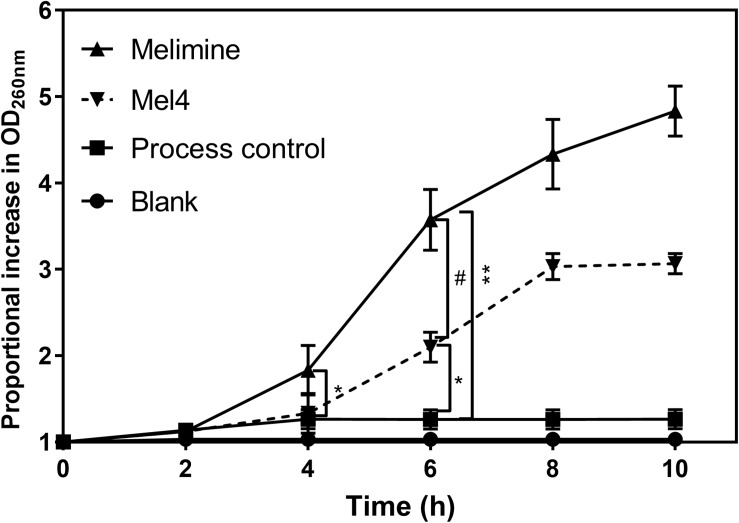
Release of nucleic acid after exposure to bound AMPs. The release of UV absorbing materials (nucleic acids) was determined spectroscopically at 260 nm in the supernatant of bacteria incubated with peptides attached surfaces. Error bars represent means (±SD) of three independent repeats in triplicate. *p = 0.005 and **p < 0.001 compared to process control while ^#^*p* < 0.001 compared to Mel4.

The release of nucleic acid from *P. aeruginosa* became significant (*p* = 0.008; Figure 7) after 4 h incubation on melimine-coated surfaces. For Mel4-coated surfaces, a significant (*p* < 0.001) increase in nucleic acid release occurred after 6 h incubation. The amount of nucleic acid increased by 4.3 ± 0.4 and 3.0 ± 0.2 times after 8 h of incubation, respectively, but began to a plateau thereafter. Surface bound melimine was associated with release of higher amounts of nucleic acid at each time point than Mel4 (*p* < 0.001). When *P. aeruginosa* 6294 was incubated with 0.297 nmole/200 μl of melimine or 0.358 nmole/200 μl of Mel4 (both equivalent to their concentrations on glass) in solution, the time at which nucleic acid was released from cells increased from 8 h to 12 h and resulted in release of 2.3 times and 1.5 times less nucleic acid, respectively, at any time pooint compared to that released by surface-immobilized peptides.

## Discussion

This study has comprehensively examined the bactericidal mechanism of two cationic peptides covalently attached to glass. Covalent attachment of the peptides on glass was confirmed with SDS treatment (Bilek and McKenzie, 2010). SDS can disrupt physical interactions such a hydrophobic binding between a protein and a surface (Bilek and McKenzie, 2010; Kondyurin et al., 2011). Both melimine and Mel4-coated surfaces lost only a small amount of amide nitrogen after SDS washing (Table 1) confirming succesfull covalent attachment of the peptides. If 11% of the melimine was released into the 200 μl of bacterial suspension, this would be equivalent of approximately 0.17 nmole/ml, and similarly 14% of Mel4 in the bacterial suspension is equivalent to appriximately 0.26 nmole/ml. These concentrations of potentially free peptide are much lower than the minimum inhibitory concentration (MIC) for melimine (66–132 nmole/ml) or Mel4 (26.6–106.5 nmole/ml) (Yasir et al., 2019) and so very unlikely to have had substantial effects on the results, indicating that the results are very likely to be due covalently attached peptide only. Previous results had confirmed that free melimine adopts a more ordered structure in a membrane mimetic environment (Rasul et al., 2010), but the confirmation of Mel4 in membrane mimetic environments had not been previously investigated. The CD spectra showed that Mel4 did not interact with vesicles containing DPPC. DPPC vesicles are non-charged lipids which mimic the mammalian cell membrane, and so the lack of interaction confirms previous data demonstrating that Mel4 was not toxic to mammalian cells in tissue culture (Dutta et al., 2016a). The vesicles composed of DMPC and DMPG would be anionic and so similar to bacterial cells membranes (Gao et al., 2012). The CD data demonstrated that the interaction of Mel4, either free or bound to quartz, with DMPC/DMPG resulted in Mel4 becoming more ordered. It is perhaps surprising that bound Mel4 behaved similarly to free Mel4. The SDS experiments had demonstrated that the vast majority of Mel4 was covalently bound to glass and so this similarity is unlikely to be the result of the spectra being derived from only free Mel4. Mel4 was covalently bound to glass using the EDC reaction, which forms amide bonds between the carboxylic acid groups of 4-azidobenzoic acid and the amine groups of Mel4. Mel4 contains amine groups at its amino terminus and on the lysine and arginine residues within its sequence. The EDC reaction is likely to randomly attach Mel4 to 4-azidobenzoic acid at any of these amine groups. Given the similarity in the CD spectra between bound and free Mel4 it appears that sufficient flexibility in Mel4 was retained after covalent binding to allow this small conformational change to occur.

Compared with the melimine-coated surface, there was less membrane permeabilization, and less ATP or nucleic acid released by the Mel4-coated surface after 4 h incubation. The lower activity of surface bound Mel4 might be driven by the smaller length of the Mel4 peptide. A smaller length may make interactions with *P. aeruginosa* membranes less favorable. Immobilized AMPs with shorter lengths of amino acids have been shown to have lower activity (Bechinger and Gorr, 2017). Moreover, Mel4 lacks tryptophan (Trp) in its sequence. Trp is hydrophobic and allows peptides to intercalate into membranes, allowing the charged resdiues to intricate with membranes (Salay et al., 2004). This in turn may induce a positive curvature in the lipid matrix which can result in displacement of membrane lipids to a greater extent (Vogel et al., 2002).

The results of the interaction of surface-bound melimine and Mel4 with *P. aeruginosa* was similar to that found with these peptides in solution (Yasir et al., 2019). However, the time course of interaction and disruption of cell membranes by surface-bound peptides was slower compared with free peptides at their minimum inhibitory concentration and usually resulted in a lower effect on the membranes. This is similar to other studies which showed that the antimicrobial peptides KLAL, MK5E, melittin, tritrpticin produced less membrane disruption (Bagheri et al., 2009, 2012) and had slower killing of *E. coli* by RK1 and RK2 following surface immobilization (Li et al., 2014). The lower bactericidal effect with slower killing kinetics of immobilized peptides might happen due to poor penetration of the peptides into membranes and less flexibility of the bound peptides. Also, the effective concentrations of the bound peptides (0.297 nmole/200 μl for melimine and 0.358 nmole/200 μl for Mel4) were substantially below the MIC for melimine (66–132 nmole/ml) or Mel4 (26.6–106.5 nmole/ml) (Yasir et al., 2019) which may also have affected the time taken for the peptides to have effects on the bacteria. Interestingly, when *P. aeruginosa* 6294 was incubated with the equivalent amount of melimine or Mel4 in solution that had been determined to be bound to glass, the time for DNA/RNA to be release was increased from 8 h to 12 h and there was less nucleic acid released. Higher activity of surface immobilized peptides compared to their soluble counterparts at the same concentration might be attributed to the availability and localized concentration of surface attached peptides for interaction with bacteria. On the other hand, exhaustion of the soluble peptides can occur after interaction with the outer membrane of the bacteria (Rai et al., 2016).

Both surface bound peptides showed interaction with the LPS of *P. aeruginosa*. Interactions between surface immobilized peptides and LPS can be mediated by electrostatic or hydrophobic forces (Farley et al., 1988; Goh et al., 2002). Mel4 has very low hydrophobic moment (0.039 μH) and melimine’s hydrophobic moment is also relatively low (0.222 μH) so it is likely their binding with LPS particularly of Mel4 is mainly mediated by charge interactions. The AMP Pep19-2.5 interacts with LPS via electrostatic binding to the phosphate group at position 4 of LPS (Kaconis et al., 2011) and so it may be that surface-bound melimine and Mel4 act in a similar manner. The lipopolysaccharide used was obtained from Sigma Aldrich and comes from *P. aeruginosa* ATCC 27316 which belongs to serotype O:10 and since immobilized melimine and Mel4 showed similar membrane interactions against different serotypes of strains used in the current study (6294, O:6; 6206, O:11; Paer1, O:1 and ATCC 19660, O:7) (Preston et al., 1991; Zhu et al., 2006), it seems unlikely that modifications (at least those that occur between these serotypes) affects the interactions of melimine or Mel4 with LPS. However, it is known that modifications to LPS such as addition of 4-aminoarabinose to the lipid A portion can result in resistance to the action of AMPs (Andersson et al., 2016). Although our initial research has shown that *P. aeruginosa* strain 6294 does not develop resistance to free melimine when passaged repeatedly for 30 days at a sub-MIC concentration (Willcox et al., 2008), future research will examine whether *P. aeruginosa* can become resistant to bound melimine or Mel4.

The mechanism of action of surface immobilized melimine and Mel4 probably begins with interaction with LPS, but then progresses to cell membrane depolarization. These results are in agreement with a previous study in which the peptide magainin attached to polyamide resins was able to cause cell membrane depolarization of *E. coli* and *S. aureus* (Haynie et al., 1995). In the current study, the membrane depolarizing effect was time-dependent but slower and demonstrated a sigmoidal kinetics compared to the hyperbolic kinetics with the soluble peptides in a previous study (Yasir et al., 2019). Cytoplasmic membrane disruption by AMPs can result in leakage of small ions first followed by release of larger cellular molecules with more profound membrane disruption (Rathinakumar et al., 2009) which is also evident by surface bound melimine and Mel4. Cytoplasmic membrane depolarization can result in leakage of impermeable substances such as ATP (Hilpert et al., 2009). Both surface bound peptides induced substantial leakage of ATP after 2 h. Similarly, in a previous study of immobilized peptides RK1 and RK2 ATP was released from *E. coli* after 2 h of exposure (Li et al., 2014). However, immobilized peptide Tet052 released ATP from *S. aureus* and *P. aeruginosa* after 30 min (Hilpert et al., 2009). The quicker release of ATP by Tet052 compared to melimine or Mel4 may have occurred due to the higher surface bound concentration (of 200 nmol) of Tet052 compared to the lower amount of bound melimine (0.297 nmol) or Mel4 (0.358 nmol) on surfaces used in the current study.

The surface immobilized peptides can permeabilize the inner membrane and release larger molecules such as nucleic acid. In the current study, membrane permeabilization to Sytox green (MW: 215.025 g/mol) that penetrates compromised membranes through reasonably large pores sizes (Makovitzki et al., 2008) started at 3 h with both the immobilized AMPs. More rapid membrane permeabilization might be achieved with higher immobilized peptides concentrations (Gao et al., 2012; Lozeau et al., 2015). Cecropin-melittin peptide immobilized onto gold nano-particles permeabilized the inner membrane of *E. coli* after 20 min at a concentration of 110 μg/cm^2^ and after 90 min at concentration of 78 μg/cm^2^ (Rai et al., 2016). The time course of release of ATP (after 2 h of exposure of bacteria to immobilized melimine or Mel4) followed by nucleic acid (after 4 h for melimine and 6 h for Mel4) probably indicates that over this time period the membranes of the bacterial cells are becoming more and more disrupted. The immobilized peptides may be forming pores in the inner membrane and those pores are becoming wider over time, therefore allowing the passage of larger molecules such as DNA/RNA. Melimine damaged the cytoplasmic membrane to a greater extent and released more nucleic acid after 4 h compared to Mel4 which released nucleic acid after 6 h, possibly due to melimine being a larger peptide and shown to be able to form an α-helix in membrane mimics (Rasul et al., 2010).

The LIVE/DEAD staining suggested that the peptide coated surfaces not only killed adherent cells but also reduced the number of total adhered cells compared to the controls. This suggests that both melimine and Mel4 may have an antifouling effect. These results are consistent with the earlier studies (Chen et al., 2009), although the mechanism of this effect is not known. It could be due dead cells being released from the coated surfaces, or that the cells are disrupted to such an extent that they do not appear in microscopic analysis. Dead cells of *P. aeruginosa* appear to be released when they adhere to surfaces coated with AMP LL-37 (Qin et al., 2015) or melimine (Dutta et al., 2016b). However, the experiments conducted with bound melimine used tritiated uridine-labeled cells, and showed that the amount of radioactivity on the surface, whilst significantly lower, was still substantial, suggesting that the inability to view cells by microscopy might be due to the total disruption of the cells.

Links between cell killing and the activity assays were investigated. Membrane depolarization resulted in rapid loss of bacterial viability of cells in a time dependent manner. In contrast to previous studies where leakage of small intracellular molecules did not affect bacterial viability (Hancock and Rozek, 2002; Mangoni et al., 2004), the current study demonstrated bacterial death was associated with release of ATP. Compared to ATP leakage, there was a higher and more rapid bacterial death during membrane depolarization. However, as bacterial cells were treated with ethylenediaminetetraacetate (EDTA) prior to exposure of immobilized peptides in the depolarization assay, this greater and more rapid effect might have been due to the presence of EDTA. EDTA can disrupt the lipopolysaccharides organization of the outer membrane of Gram-negative bacteria by displacing metal ions (Mg^+2^ and Ca^+2^) so facilitate immobilized peptides to disrupt inner membranes (Hilpert et al., 2009).

## Conclusion

In conclusion, similar to solution phase, both immobilized melimine and Mel4 were able to bind bacterial LPS, could cause membrane disruption, facilitated release of ATP and then DNA/RNA from cells, and this was associated with death of adherent *P. aeruginosa*. This indicated that the cell membrane is still an important target for surface immobilized AMPs. However, the membrane disruption and killing kinetics of immobilized AMPs occurred at a slower rate compared to their soluble counterparts when these were used at the minimum inhibitory concentration. Notwithstanding this, at similar concentration to immobilized peptides, soluble peptides did not release any DNA/RNA at 4 h. In contrast, surface immobilized melimine and Mel4 disrupted the membrane to larger extent by releasing 2.3 times and 1.5 times nucleic acid than their soluble counterparts at the same concentration after 8 h of incubation.

## Data Availability Statement

All datasets generated for this study are included in the article/Supplementary Material.

## Author Contributions

MY designed the study, performed the experiments, and wrote the manuscript. KRH, RC, KKH, RJC, and RK helped in performing the experiments and analyzing the data. NK and MW planned the project and developed the theoretical framework. MW and DD edited the manuscript.

## Conflict of Interest

The authors declare that the research was conducted in the absence of any commercial or financial relationships that could be construed as a potential conflict of interest.

## Footnotes

1 https://capito.uni-jena.de
