# Supplementary material for: Mechanism of Action of Surface Immobilized Antimicrobial Peptides Against Pseudomonas aeruginosa
Source: Front Microbiol. 2020 Jan 22;10:3053. doi: 10.3389/fmicb.2019.03053 (PMC6987417; doi:10.3389/fmicb.2019.03053)
Supplement: Supplementary file 1 [file Data_Sheet_1.PDF]

Supplementary Material

Table S1. Cytoplasmic membrane depolarization of *P. aeruginosa* by surface attached peptides and control surfaces. Cell membrane depolarization was assessed by the release of the membrane potential sensitive dye DiSC3-5, measured spectroscopically at 622_{nm} excitation and 670_{nm} emission wavelengths. Data presented as means (±SD).

Time (h)	*P. aeruginosa* 6206				*P. aeruginosa* Paer1				*P. aeruginosa* ATCC 19660			
	**Melimine**	**Mel4**	**Process control**	**Blank**	**Melimine**	**Mel4**	**Process control**	**Blank**	**Melimine**	**Mel4**	**Process control**	**Blank**
**0**	0±0	0±0	0±0	0±0	0±0	0±0	0±0	0±0	0±0	0±0	0±0	0±0
**15**	22±3	16±5	1±1	1±0	14±2	13±4	1±1	1±0	16±4	13±4	3±1	1±1
**30**	27±9	22±3	2±1	1±0	22±4	19±3	2±1	1±0	20±5	19±3	3±1	1±1
**45**	32±9	25±5	2±1	1±1	30±4	23±3	2±1	1±1	26±4	23±3	3±1	2±1
**60**	42±3	36±4	2±1	1±1	36±7	32±3	2±1	1±1	37±2	32±3	2±2	2±1
**75**	47±7	40±5	2±2	1±1	41±8	37±3	2±2	1±1	40±5	37±3	2±2	2±1
**90**	50±3	43±6	2±2	1±1	49±5	43±3	2±1	1±1	46±3	43±3	2±2	2±1

Table S2. Corresponding death of *P. aeruginosa* due to cytoplasmic membrane depolarization by surface attached peptides. Data presented as means (±SD).

Time (h)	*P. aeruginosa* 6206				*P. aeruginosa* Paer1				*P. aeruginosa* ATCC 19660			
	**Melimine**	**Mel4**	**Process control**	**Blank**	**Melimine**	**Mel4**	**Process control**	**Blank**	**Melimine**	**Mel4**	**Process control**	**Blank**
**0**	0±0	0±0	0±0	0±0	0±0	0±0	0±0	0±0	0±0	0±0	0±0	0±0
**15**	60±14	70±42	3681±1	280±1113	43±31	130±44	300±87	322±106	43±31	130±44	300±87	322±106
**30**	65±21	85±49	560±85	580±113	113±42	210±85	612±97	603±96	113±42	203±75	612±97	603±96
**45**	75±35	75±35	765±49	720±113	157±40	263±71	743±81	773±64	157±40	220±70	743±81	773±64
**60**	57±4	85±49	900±141	865±163	193±70	293±85	910±96	923±108	193±70	250±62	910±96	923±108
**75**	68±17	170±42	1120±170	1100±141	250±50	333±104	1127±110	1110±184	243±51	283±76	1127±110	1143±212
**90**	110±28	215±21	1420±170	1335±119	273±87	433±126	1390±165	1380±171	273±87	367±61	1390±165	1413±186

Table S3. The leakage of cellular ATP in nano moles (nmoles) following incubation of bacteria with AMPs attached and control surfaces. Data presented as means (±SD).

Time (h)	*P. aeruginosa* 6206				*P. aeruginosa* Paer1				*P. aeruginosa* ATCC 19660			
	**Melimine**	**Mel4**	**Process control**	**Blank**	**Melimine**	**Mel4**	**Process control**	**Blank**	**Melimine**	**Mel4**	**Process control**	**Blank**
**1**	0.2±0.1	0.2±0.1	0.2±0.1	0.1±0.1	0.2±0.1	0.2±0.1	0.2±0.1	0.1±0.1	0.2±0.1	0.2±0.1	0.2±0.1	0.1±0.1
**2**	1.4±0.3	1.2±0.3	0.2±0.1	0.1±0.1	0.9±0.3	0.8±0.4	0.2±0.1	0.1±0.1	1.5±0.4	1.3±0.2	0.2±0.1	0.1±0.1
**4**	2.4±0.3	1.4±0.3	0.2±0.1	0.1±0.1	1.6±0.3	1.2±0.2	0.2±0.1	0.1±0.1	2.5±0.5	1.6±0.3	0.2±0.1	0.1±0.1

Table S4. Inner membrane permeabilization by surface attached peptides. Fluorescence intensity due to interaction of Sytox green dye with DNA following incubation with peptides attached surfaces was measured spectroscopically at 480_{nm} excitation and 522_{nm} emission wavelengths. Data presented as means (±SD).

Time (h)	*P. aeruginosa* 6206				*P. aeruginosa* Paer1				*P. aeruginosa* ATCC 19660			
	**Melimine**	**Mel4**	**Process control**	**Blank**	**Melimine**	**Mel4**	**Process control**	**Blank**	**Melimine**	**Mel4**	**Process control**	**Blank**
**1**	1±0	1±0	1±0	1±0	1±0	1±0	1±0	1±0	1±0	1±0	1±0	1±1
**2**	2±1	2±1	3±2	2±1	2±1	1±1	1±0	1±0	2±1	1±1	5±3	1±2
**3**	55±10	24±7	7±4	3±2	46±13	28±7	2±1	1±1	43±8	23±9	5±3	2±2
**4**	127±25	49±9	7±4	3±3	85±23	40±14	2±0	1±0	93±25	34±14	6±4	2±2

Table S5. Percentage (%) coverage of Live and Dead cells on peptides coated and control surfaces. Fluorescence microscopy captured images were examined for live/dead bacteria with the help of image J software.

Surfaces	***P. aeruginosa* 6206**		***P. aeruginosa* Paer1**		***P. aeruginosa* ATCC 19660**	
	**% Live cells**	**% Dead cells**	**% Live cells**	**% Dead cells**	**% Live cells**	**% Dead cells**
**Blank**	97±5	3±2	97±8	3±2	97±0	2±1
**Process control**	94±5	6±4	98±5	2±1	98±0	3±2
**Melimine**	37±3	63±17	48±16	52±4	34±2	66±16
**Mel4**	53±23	48±18	55±13	45±8	60±12	40±12

Table S6. Percentage (%) inhibition of bacterial attachment by peptides coated surfaces compared to control as analyzed through image J software.

***P. aeruginosa* 6206**			***P. aeruginosa* Paer1**			***P. aeruginosa* ATCC 19660**		
**Process control**	**Melimine coated**	**Mel4 coated**	**Process control**	**Melimine coated**	**Mel4 coated**	**Process control**	**Melimine coated**	**Mel4 coated**
**100±4**	57±9	44±18	100±6	66±9	64±13	100±3	54±9	51±10

Table S7. Proportional increase of DNA/RNA as determined spectroscopically by increasing OD 260nm. Data presented as means (±SD).

Time (h)	*P. aeruginosa* 6206				*P. aeruginosa* Paer1				*P. aeruginosa* ATCC 19660			
	**Melimine**	**Mel4**	**Process control**	**Blank**	**Melimine**	**Mel4**	**Process control**	**Blank**	**Melimine**	**Mel4**	**Process control**	**Blank**
**0**	1.0±0.0	1.0±0.0	1.0±0.0	1.0±0.1	1.0±0.0	1.0±0.0	1.0±0.0	1.0±0.1	1.0±0.0	1.0±0.0	1.0±0.0	1.0±0.1
**2**	1.4±0.2	1.2±0.1	1.1±0.2	1.0±0.1	1.2±0.1	1.2±0.1	1.1±0.1	1.0±0.1	1.2±0.2	1.1±0.2	1.1±0.2	1.0±0.1
**4**	1.6±0.3	1.4±0.4	1.3±0.1	1.0±0.1	1.5±0.4	1.5±0.4	1.2±0.2	1.0±0.1	1.8±0.3	1.4±0.5	1.1±0.1	1.0±0.1
**6**	2.7±0.4	2.6±0.5	1.3±0.1	1.0±0.1	2.8±0.4	2.1±0.2	1.2±0.3	1.0±0.1	2.6±0.2	2.3±0.5	1.2±0.2	1.0±0.1
**8**	3.9±0.2	2.9±0.3	1.3±0.1	1.0±0.1	3.8±0.1	3.4±0.3	1.3±0.3	1.0±0.1	3.3±0.3	2.6±0.4	1.2±0.2	1.0±0.1
**10**	3.9±0.2	3.0±0.3	1.3±0.1	1.0±0.1	4.0±0.1	3.5±0.4	1.3±0.3	1.0±0.1	3.4±0.3	2.7±0.5	1.2±0.2	1.0±0.1
